# *ARAF* mutated langerhans cell histiocytosis developed on the background of *ARAF*/*JAK2* mutated polycythaemia vera: evidence of branching evolution from the same progenitor cell. A case report and literature review

**DOI:** 10.1007/s00277-026-07168-w

**Published:** 2026-07-15

**Authors:** Gorana Gašljević, Panna Dora, Biljana Grčar-Kuzmanov, Tereza Urdih, Sandra Šućurović, Helena Podgornik, Vida Stegel, Matjaž Sever

**Affiliations:** 1 Department of Pathology, Institute of Oncology Ljubljana, Ljubljana, Slovenia; 2 Faculty of Medicine, University of Maribor, Maribor, Slovenia; 3 Department of Haematology, University Clinical Centre Ljubljana, Ljubljana, Slovenia; 4 Faculty of Pharmacy, University of Ljubljana, Ljubljana, Slovenia; 5 Department of Molecular Diagnostics, Institute of Oncology Ljubljana, Ljubljana, Slovenia; 6 Faculty of Medicine, University of Ljubljana, Ljubljana, Slovenia

**Keywords:** Langerhans cell histiocytosis, Polycythaemia vera, *ARAF* mutation, *IDH2* mutation, Divergent evolution

## Abstract

The case report presents the first documented instance of *ARAF*-mutated Langerhans cell histiocytosis (LCH) arising in a patient with *ARAF*/*JAK2*-mutated polycythaemia vera (PV), providing compelling evidence for a clonal relationship and branching evolution from a common progenitor precursor. The patient, a 63-year-old male, initially presented with classic PV symptoms and was diagnosed with *JAK2* p.V617F mutated PV. Over the disease course, persistent monocytosis and progressive splenomegaly were noted, with subsequent development of a diffuse, non-itching maculopapular skin eruption in the setting of LCH. Histopathological and molecular analyses of bone marrow and skin lesions revealed the presence of *ARAF*, *IDH2* and *ASXL1* mutations in both tissues. This molecular profile supports the hypothesis of divergent evolution from a shared *ARAF/IDH2/ASXL1*-mutated clone, with subsequent acquisition of *JAK2*. Therapeutic management included phlebotomies, aspirin, hydroxyurea, peginterferon alfa-2a, and ruxolitinib, with variable responses and eventual disease progression. The patient ultimately succumbed to infectious complications. We further reviewed the literature on clonally related myeloproliferative neoplasms and LCH, highlighting the rarity of such cases and the potential role of chronic inflammation, monocytosis, and targeted therapies (e.g., JAK inhibitors) in disease evolution. The findings underscore the importance of comprehensive molecular profiling in patients with overlapping haematologic and histiocytic disorders and propose a model of branching evolution that may inform future research and therapeutic strategies. This first reported case expands the understanding of LCH ontogeny and its association with myeloid neoplasms, emphasizing the need for further studies on clonal haematopoiesis and lineage plasticity in these rare disease intersections.

## Background

Langerhans cell histiocytosis (LCH) is a clonal neoplasm of myeloid dendritic cells that have an immunophenotype of Langerhans cells expressing CD1a and CD207 and showing Birbeck granules by ultrastructural examination; high-grade cytologic features are characteristic for Langerhans cell sarcoma (LCS) and are absent in LCH [1]. It is a rare disorder, more frequently affecting the paediatric population [2]. According to the number of organs or systems involved, LCH could be clinically classified into a single-system LCH (SS-LCH) which occurs in one organ system and can be unifocal or multifocal, and multisystemic LCH (MS-LCH) which occurs in two or more organ systems. MS-LCH with bone marrow, spleen, or liver involvement is considered a high-risk subtype of LCH [3]. Gene mutations of the MAPK pathway play an essential role in LCH pathogenesis [3]. They are present in majority of cases with consequent expression of phosphorylated ERK. According to the proposed misguided-myeloid differentiation model of LCH ontogeny [3], the clinical phenotype depends on the level of stem cell differentiation at which MAPK pathway mutations occur. The most prevalent mutation is *BRAF* p.V600E, followed by *MAP2K1* mutations, while *ARAF* mutations are very rare [4]. *ERBB3* and *N/KRAS* mutations have rarely been reported in adult patients [3]. Approximately 10% of LCH arise in association with other haematological neoplasms, which commonly entail LCH-AHN (LCH-associated with other haematological neoplasm) [2]. Most frequently, chronic myelomonocytic leukaemia (CMML) is present in these patients, sometimes myelodysplastic syndrome (MDS) and rarely myeloproliferative neoplasm (MPN). To date, there are only very rare case reports [5–7] about well documented clonally related primary myelofibrosis (PMF) and LCH, while development of clonally related LCH in polycythaemia vera (PV) patient has never been reported.

PV is a type of *BCR::ABL1*-negative MPN, characterized by erythrocytosis, that is frequently accompanied by leucocytosis and/or thrombocytosis. It is typically linked to activating *JAK2* mutations, with *JAK2* p.V617F being the most common one. Most commonly, it presents with fatigue, aquagenic pruritus, night sweats, symptoms that are consequence of hyperviscosity or with splenomegaly-related symptoms. PV is a relatively indolent disease; however, it can undergo leukemic transformation in 2–15% of patients or it can progress to a post-PV-myelofibrosis [8, 9]. Certain cytogenetic abnormalities [10] and mutations [11] correlate with leukemic transformation. A poorer prognosis of PV patients with higher leukemic transformation rate was described in PV patients with monocytosis as well [12].

Here we report extremely rare concomitant PV and secondary clonally related LCH with skin involvement. We describe their molecular characteristics and propose a model of branching evolution from the same progenitor cell for the first time. Furthermore, we provide a review of all clonally related MPN and LCH cases reported.

Case report.

**Fig. 1:**
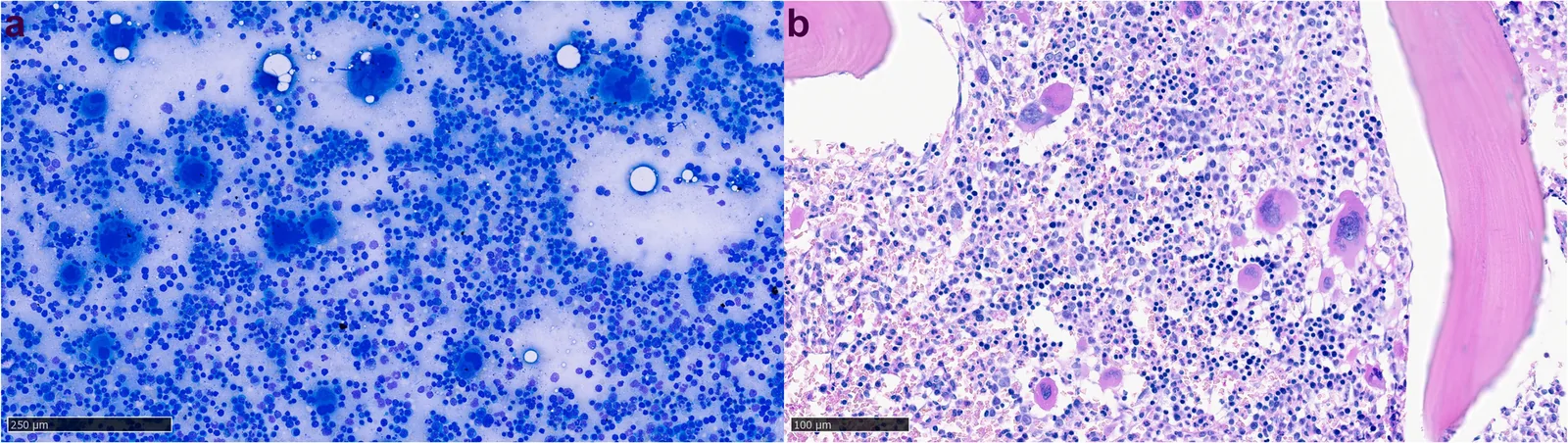
A May Grunwald Giemsa (MGG) stained initial BM aspirate; B H&E of initial BM trephine biopsy, 20x

A 63-year-old Caucasian male, otherwise healthy and without significant medical history, presented in July 2021 with tinnitus, blurred vision, aquagenic pruritus and night sweats. The liver and spleen were palpable at examination. Haematological work up showed an increased erythrocyte number of 8.22 × 10^12^/L, as well as elevated haemoglobin (Hb) and haematocrit (Hct) levels of 167 g/L and 0.568 respectively. The leukocyte and platelet counts were normal (9.71 × 10^9^ and 297 × 10^9^, respectively). Additionally, absolute monocytosis (2.92 × 10^9^/L) was present in the differential blood count. Serum erythropoietin level was low, being < 2.5 U/L. Abdominal ultrasound showed splenomegaly of 19 cm and hepatomegaly of 16 cm. Cytogenetics from bone marrow (BM) aspirate revealed normal karyotype (46, XY). From the peripheral blood (PB), *JAK2* p.V617F mutation was identified by polymerase chain reaction (PCR). First BM aspirate and trephine biopsy showed hypercellular BM with trilinear hyperplasia, normal maturing erythropoiesis, slightly left-shifted granulopoiesis, along with an increased number of megakaryocytes being distributed in clusters (Fig. 1A, B). Majority of the megakaryocytes showed a myeloproliferative morphology, while partially being dysplastic as well. Immunohistochemical staining for CD14 revealed an increased monocytic population in the BM. There was no increase in in blast cell numbers. Reticulin fibrosis was at the time only mild (MF-1) and there was no collagen fibrosis.

**Fig. 2:**
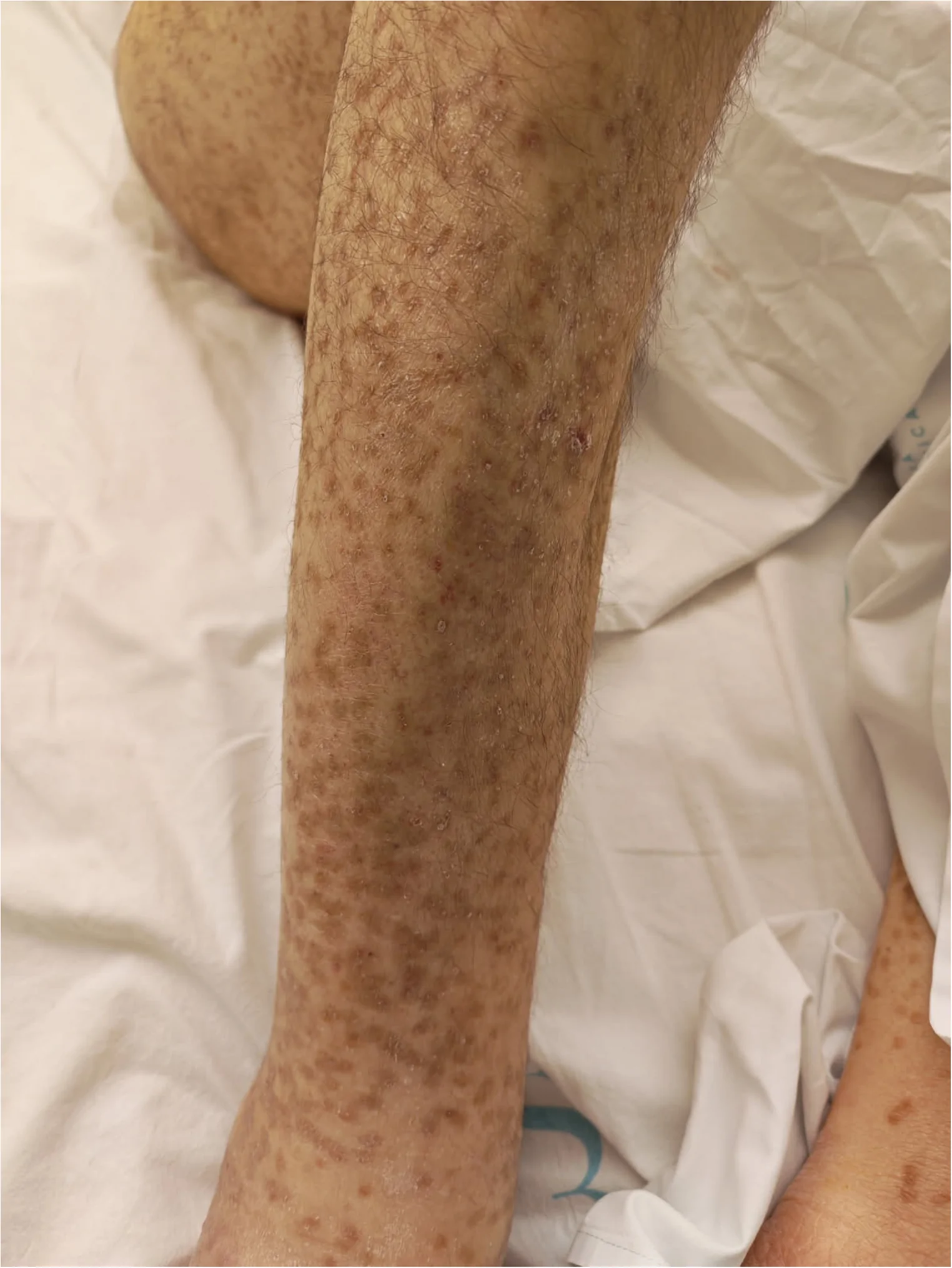
Diffuse maculopapular skin eruption (leg)

The patient started with Aspirin and phlebotomies. Initial therapy with phlebotomies was repeatedly complicated by orthostatic hypotension and had to be stopped. Cytoreductive therapy with hydroxyurea (HU) was introduced. The Hct was controlled on HU for six months when persistent mild neutropenia and thrombocytopenia developed in spite of dose adjustments. Patient was switched to peginterferon alfa-2a (IF). With this treatment, adequate Hct and Hb levels were maintained, cytopenias improved but absolute and relative monocytosis persisted. After six months on IF he developed night sweats, weight loss and worsening of splenomegaly. IF was stopped and he was started on ruxolitinib. The initial low dose ruxolitinib had to be increased to 20 mg BID to control symptoms and Hct. After 19 months on ruxolitinib, neutropenia and thrombocytopenia developed with loss of spleen response. In May 2024, a persistent, non-itching diffuse maculopapular skin eruption developed (Fig. 2).

**Fig. 3:**
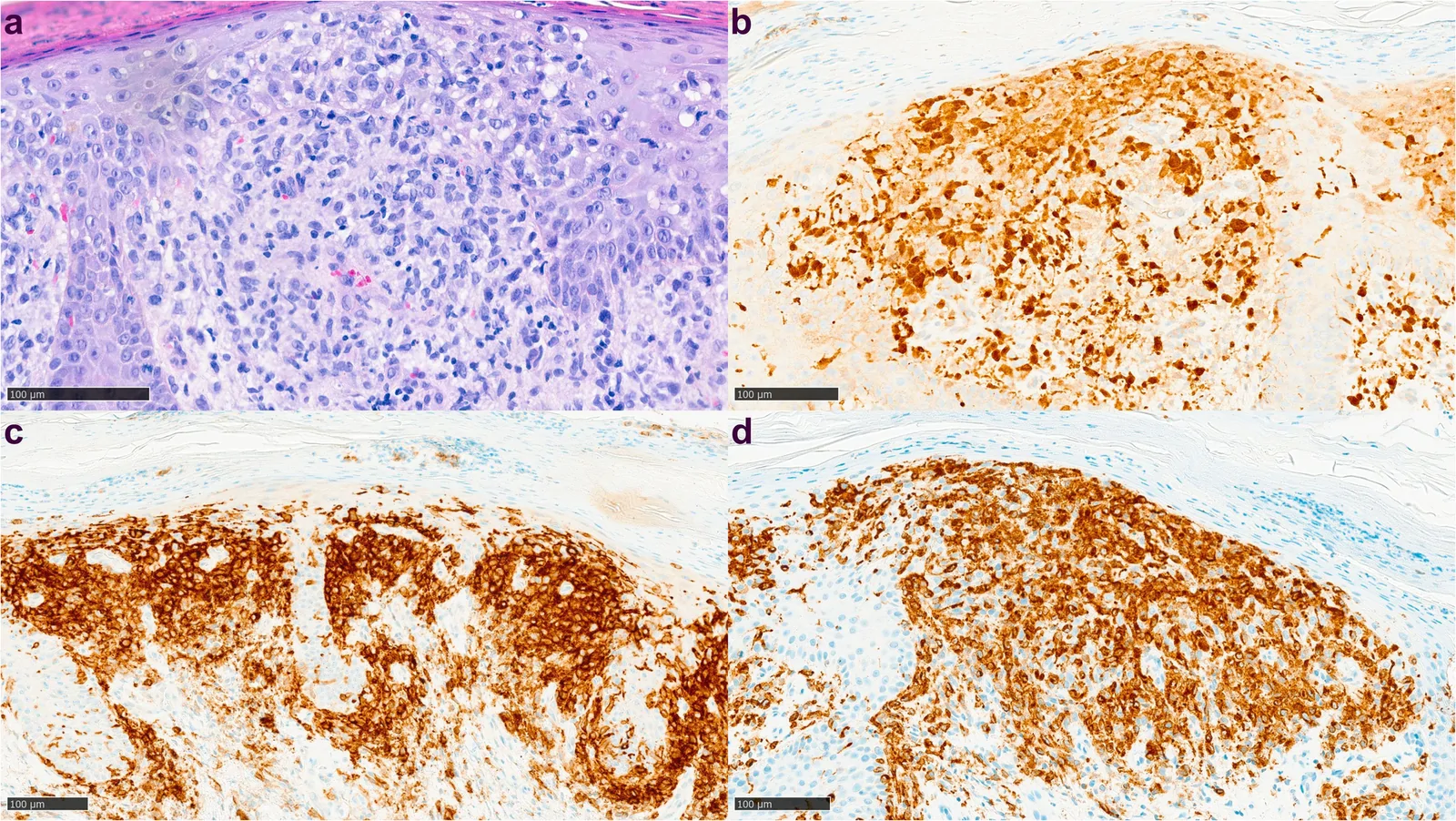
Skin biopsy: A H&E, 20x; B S-100, 5x; C CD1a, 5x; D Langerin (CD207), 5x

Skin biopsy showed a band-like infiltrate in the upper dermis, consisting of medium sized cells with indented, grooved nuclei and indistinct eosinophilic cytoplasm that were immunohistochemically positive for CD1a, CD207 and S100, and negative for CD163 (Fig. 3A-D).

**Fig. 4:**
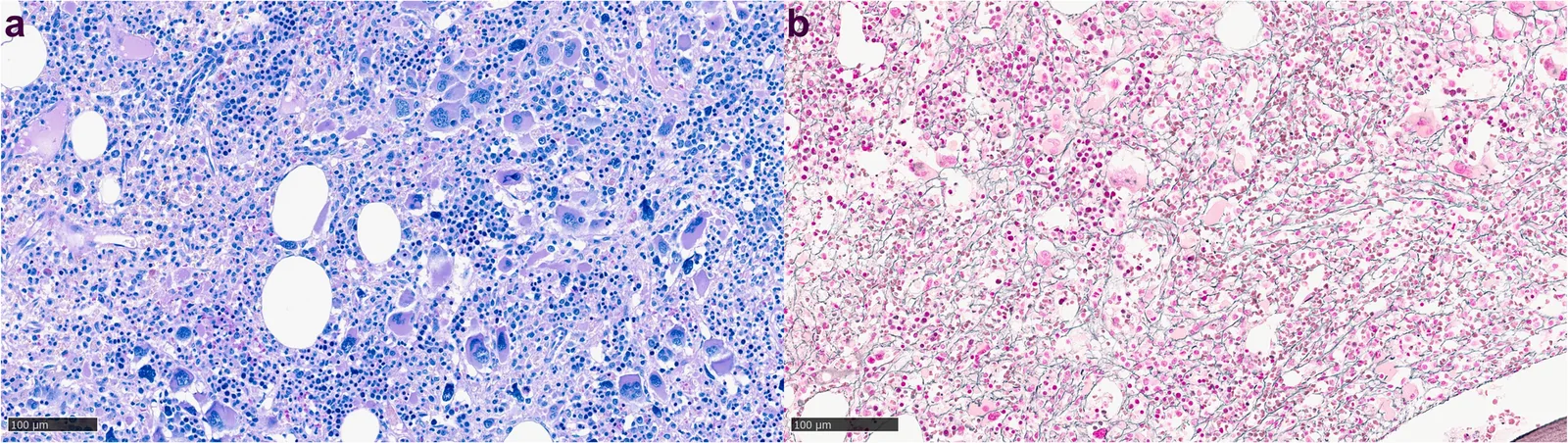
BM trephine from the time of skin eruption development; A H&E, 20x; B Gomori staining showing reticuline fibrosis of grade 3; 20x

In August 2024, repeated BM trephine showed panmyelosis with left shifted granulopoiesis and a more hyperplastic megakaryopoiesis in comparison to the 2021 biopsy (Fig. 4A). There was no increase in blast cells. However, reticulin fibrosis was of grade III (MF-3) (Fig. 4B) and collagen fibrosis of grade I (CF-1).

Additional molecular analysis was performed by next generation sequencing in the BM and skin specimens.

**Fig. 5:**
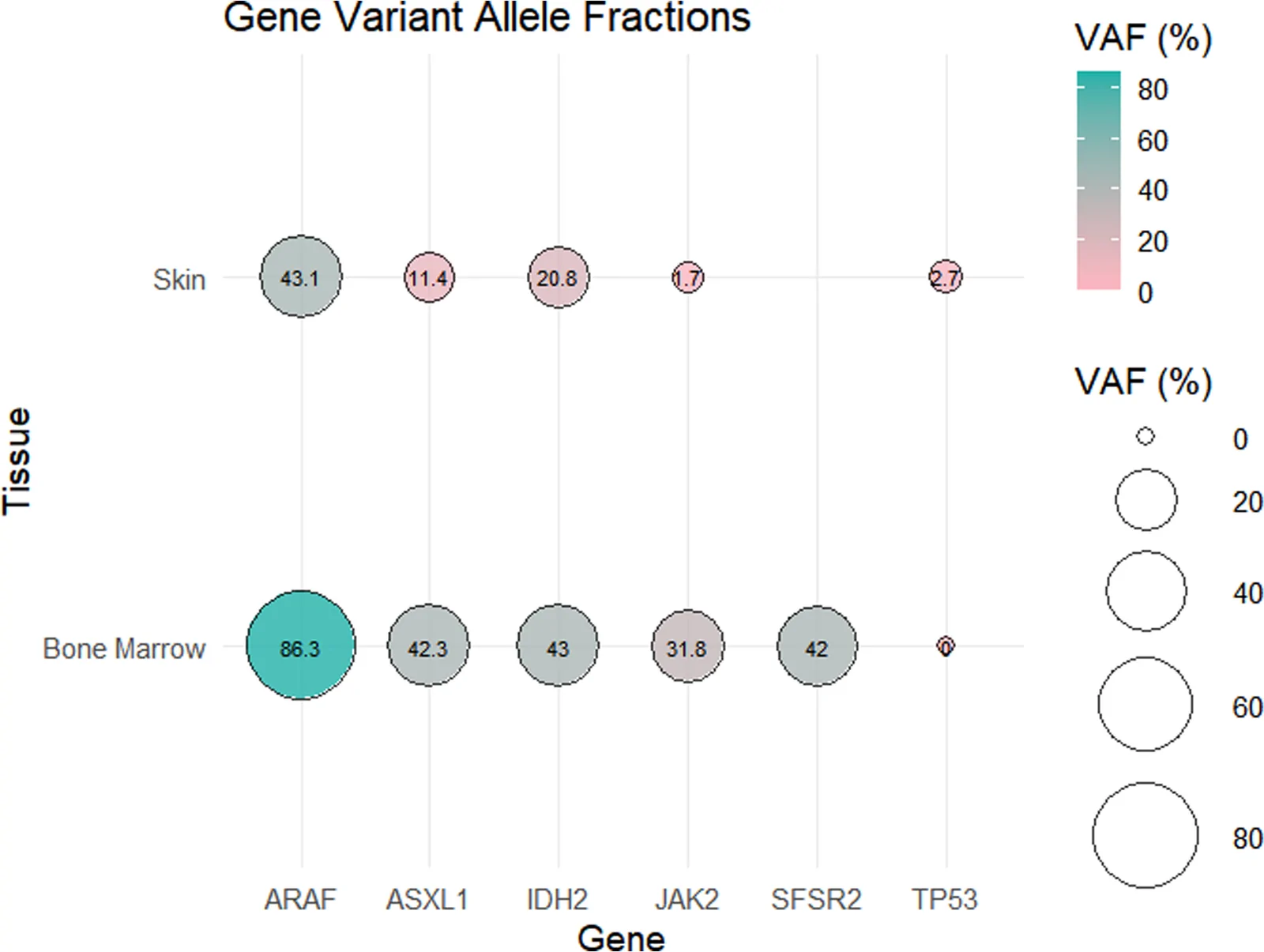
Bubble plot showing distribution of the mutation based on Archer VariantPlex Myeloid, Oncomine Precision Assay GX and TruSight Oncology 500 in bone marrow and skin specimens

In the BM, NGS (Archer VariantPlex Myeloid) revealed pathogenic variants in genes *ASXL1* p.(Leu775Ter) NM_015338.5:c2324delT with variant allelic frequency (VAF) 42.3%, *SRSF2* NM_001195427.1:c.284 C > G p.(Pro95Arg) with VAF 42% and *IDH2* p.(Arg 140Gln) NM_002168.2:c.419G > A with VAF 43% beside already known *JAK2* p.(Val617Phe) NM_004972.3:c.1849G > T with VAF 31.81%. Additionally, out of the routine diagnostic procedure BM aspirate was tested by Oncomine Precision Assay GX (ThermoFisher Scientific) revealing *ARAF* p.(ser214Phe) NM_001654.5:c.641 C > T with VAF 86.3% (Fig. 5).

In the skin (Oncomine Precision Assay, ThermoFisher Scientific), the same *IDH2* c.419G > A p.(Arg 140Gln) with VAF 20.8% was present and besides *ARAF* c.641 C > T p.(Ser21Phe) VAF 43.1% was identified. Similar to the BM specimen, out of the routine diagnostic procedure skin specimen was additionally tested by TruSight Oncology 500 revealing the same *ASXL1* mutation as was present in the BM with VAF 11.45%. *JAK2* c.1849 G > T p.(Val617Phe) was present as well but with very low VAF of only 1.71% as well as *TP53* p.Arg248Trp NM_000546.5:c.742 C > T with VAF 2.74% (Fig. 5).

Therapy with ruxolitinib was continued in spite of moderate cytopenias as the patient still had a clinical response. Over time, a slow and gradual increase in hepatosplenomegaly was observed. In July 2025 the patient was presented to a multidisciplinary team for potential bone marrow transplant; however, his age, moderately impaired pulmonary function and progressive splenomegaly would have precluded the procedure. In December 2025 the patient suffered a minor trauma to his upper left extremity that resulted in a haematoma, which became infected. He was treated in hospital but the infection progressed to eventual sepsis and multiorgan failure, leading to the death of the patient.

## Discussion

During their ontogenesis, haematopoietic stem cells, among others, differentiate towards the cells of the mononuclear phagocytic system, which includes monocytes, macrophages, and dendritic cells [13]. These cell types show extensive overlap in their properties and functions in the immune response; hence, circulating monocytes can enter tissues to differentiate into effector cells, including macrophages and dendritic cells (DC) [13]. Human DC are classified into two groups; myeloid (mDC, which are further subdivided into mDC1 and mDC2) and plasmacytoid (pDC) dendritic cells. Langerhans cells are DC localized in mucosal linings, epidermis and bronchial epithelium. Under normal conditions, they are self-renewing. However, in diseases like MPN, that are characterized by an inflammatory milieu and increased myeloid cell proliferation, monocytes could undergo differentiation into LC [14].

Most histiocytic/dendritic cell neoplasms arise de novo; however, there is a subset of cases that develops in association with other haematological neoplasms (AHN) [2]. They often bear genetic alterations which at least partially overlap with AHN, suggesting that a divergent differentiation occurs from a common progenitor clone that is either lymphoid or myeloid [5, 6, 15, 16].

Skin infiltration in the setting of myeloid neoplasms can be seen in approximately 10% of all patients [17] and is most frequently in the form of leukaemia cutis (granulocytic sarcoma), or in the form of leukaemia cutis-like lesions (mature myelomonocytic infiltrates in CMML, CML, MDS) [17]. Cutaneous involvement in Ph-negative MPN is very rare. In the large review study performed by Bonometti [18] covering the time span of almost 90 years, only 43 papers with 47 patients being reported were identified. The vast majority of those patients did have infiltrates of extramedullary haematopoiesis or leukaemia cutis, while in 10% of patients, histiocytic proliferations were identified, among them some rare patients with LCH. All of those patients had PMF. So far, secondary skin LCH has been described and molecularly characterized in three patients with PMF (Table 1). Table includes two well documented cases of LCH and one case with partial Langerhans phenotype, because the results of the CD207 immunostaining were questionable [6].

**Table 1:** Review of the literature on documented clonally related MPN and LCH

AUTHOR YEAR	Bonometti 2018	Holst 2021	Isei 2023	Present case 2025
SEX AGE	Female 70	Male 69	Female 65	Male 63
MPN	PMF	PMF	PMF	PV
HISTIOCYTOSIS	LCH	LCH vs. ICH	LCH	LCH
HISTIOCYTIC IMMUNOPHENOTYPE	S100/CD1a/CD207+	S100/CD1a+/CD207q	CD1a/CD207+	S100/CD1a/CD207+
MPN MUTATION	*JAK2 V617F*	*JAK2 V617F*	*CALR1*	*JAK2 V617F* *ARAF* *IDH2* *ASXL1* *SRSF2*
DRIVER AND CH MUTATIONS IN HISTIOCYTOSIS	*JAK2 V617F*	*JAK2 V617F*	*CALR1*	*ARAF* *IDH2* *ASXL1*
ADDITIONAL MUTATIONS IN HISTIOCYTOSIS	*BRAF* WT	*BRAF* +	*BRAF* WT	*BRAF* WT
SYSTEM INVOLVEMENT	MC MF	MC MF	MC MF	MC MF MS-LCH can not be ruled out
RUXOLITINIB AND TIME TO HISTIOCYTOSIS DEVELOPMENT	Yes 7 months	NA	Yes 48 months	Yes 18 months
OUTCOME (months from dg of histiocytosis)	DOD 31 months	DOD 12 months	DOD 10 months	DOD 16 months
MONOCYTOSIS	No	No	No	Yes Absolute and Relative

Legend: *MPN* myeloproliferative neoplasm, *PMF* primary myelofibrosis, *PV* polycythemia vera, *LCH* Langerhans cell histiocytosis, *ICH* Indeterminate cell histiocytosis, q questionable, *WT* wild type, *MC* mucocutaneous, *MF* multifocal, *NA* not available, *DOD* died of disease

All patients were diagnosed with PMF. In two of three cases, *JAK2* V617F mutation was present and one was *CALR* mutated. In our case PV was *JAK2* V617F mutated with additional mutations in *ARAF*, *IDH2*, *ASXL1* and *SRSF2.* In all three reported PMF cases with driver mutations present, the same mutation was revealed in LCH as well. In the present case, LCH carried the same *ARAF*,* ASXL1* and *IDH2* mutations but not the driver mutation. Additionally, in one case the LCH population had a *BRAF* mutation. Hence, in three out of four cases, the clonal relationship between MPN and LCH was undoubtedly proven. Two out of three, and additionally our patient, were treated with ruxolitinib and developed skin lesions in the time span of 7–48 months. All patients had mucocutaneous involvement; one with single lesion, all others with multiple. Bonometti et al. [19] tried to investigate the possible pathogenic mechanism underlying the association of the two rare diseases. They proposed a model in which clonal CD1a+ proliferation was influenced and supported by sustained chronic inflammation with increased GM-CSF and elevated IL-4 and/or TGF-β on one side, and by JAK1/2 blockage by ruxolitinib on the other. JAK1/2 blockage leads to the inhibition of STAT5 activation and consequently to the stimulation of CD1a + DC proliferation. The development of LCH-SS in reported patients with MPN is very much in line with the proposed misguided-myeloid-differentiation model of LCH ontogeny [3]. Based on this, the stage of differentiation in which the myeloid cell acquires activating *MAPK* mutations determines the extent and clinical phenotype of LCH. However, in the present case *ARAF* mutation was present with the high VAF already in the BM. Since *ARAF* is an X-linked gene and is hemizygous in male patient, high *ARAF* VAF indicates mutations on the level of stem cell/common myeloid precursor very probably being embedded within the dominant *IDH2/ASXL1/SFSR2* mutated clone. Mutations that activate RAS-RAF-MEK-ERK pathway are rare and only occasionally described in MPN [20, 21]. These mutations are primarily subclonal, frequently indicating disease progression and leukemic transformation [22]. Inhibition of JAK/STAT pathway in MPNs cause compensatory overactivation of MAPK pathway and confers a strong selective growth advantage of pre-existing RAS/RAF mutated clones representing a powerful downstream driver, increasing their cellular fitness and promoting more aggressive disease [23], characterized by prominent leukocytosis, higher platelet counts, and worsening splenomegaly. Our patient presented with cutaneous LCH involvement; however, since ARAF mutation presumably developed in early, common myeloid precursor cell and there was no evaluation of LCH in other organs (spleen, liver), existence of MS-LCH cannot be completely ruled out. The lower VAFs of *IDH2* and *ARAF* in the skin (approximately half of those observed in the bone marrow) are most likely a consequence of sample dilution, as a significant amount of normal background tissue (dermis, epidermis, and blood vessels) was present. *ASXL1* was found in skin with VAF 11.45% at the point when paraffin block of the skin biopsy was already exhausted with smaller amount of residual tumour tissue. Besides, *TP53* mutation with VAF 2.74% and *JAK2* with VAF 1.71% were revealed in the skin, *TP53* most probably representing subclonal mutation while *JAK2* more probably represents contamination from the peripheral blood than subclonal mutation. Furthermore, Sadeghian et al. [24] reported a case of *BRAF* positive Langerhans cell sarcoma (LCS) arising from *CALR* positive MPN with evidence of a clonal relationship. NGS was performed on skin and BM biopsies revealing several shared mutations: *ASXL1* (p. G646fs), *CALR* (p. L367fs), and *TET2* (p.Y1618*). These findings confirmed a relationship between LCS and the patient’s underlying MPN.

**Fig. 6:**
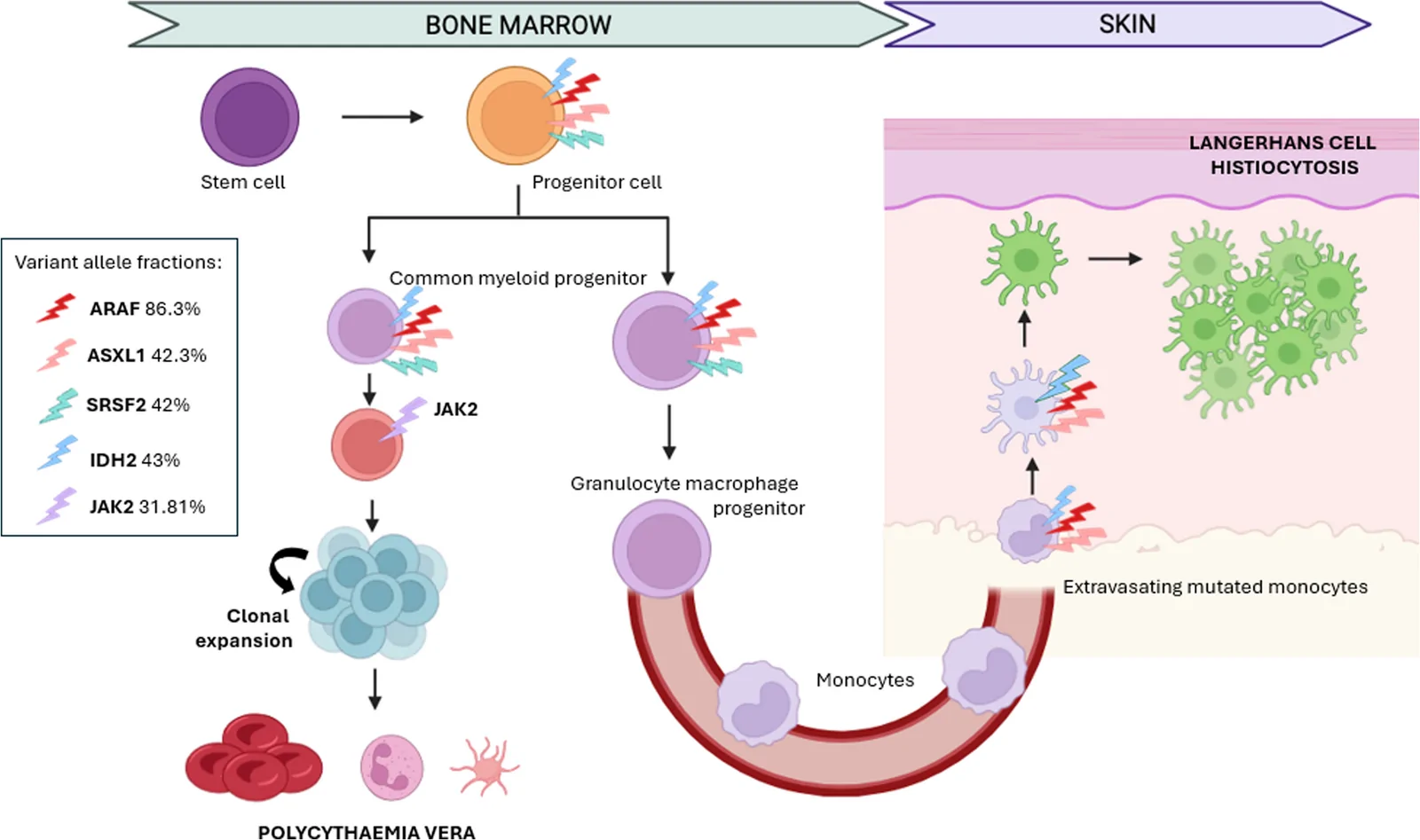
Proposed model of branching evolution of PV and LCH from the common *ARAF/IDH2/ASXL1* mutated clone. Image was generated using BioRender

We are therefore proposing a model of branching evolution of PV and LCH from the common *ARAF/IDH2/ASXL1* mutated clone (Fig. 6). Additionally, (Fig. 6) shows step by step development of PV, as suggested by Luque Paz et al. [25].

We considered monocytosis to be an adverse factor for the development of subsequent LCH, since our patient showed CMML-like phenotype from the very beginning of the disease. In addition, monocytosis was present when LCS developed following MPN [24]; of note, authors excluded CMML-like progression of PMF. No data was found about monocytosis in other reports. Consequently, more reports with focus on existing monocytosis are needed to confirm the assumption that a CMML-like phenotype could represent an adverse factor for the development of secondary LCH/LCS by representing a reservoir of potential LC precursors.

The treatment of the patient was planned as PV management from the beginning. Phlebotomies and Aspirin are the most common first-line approach. Though rare, complications of phlebotomies in PV, such as orthostatic hypotension, were observed in clinical practice [26]. Hydroxyurea is standard of care for elderly patients; however, poor tolerance can be seen with possible side effects or acquired resistance occurring [27]. In our case cytopenias, in spite of dose adjustment, precluded further HU therapy. We decided to proceed with interferon-alfa as the preferred option among approaches to manage PV in younger patients [28, 29]. This improved the patient’s cytopenias but the PV response was shorter than expected. Ruxolitinib was the remaining choice of treatment and it controlled the disease well until the last months of the follow-up [30, 31]. The initial requirement for a relatively high ruxolitinib dose (15–20 mg BID), together with the subsequent emergence of cytopenias and progressive splenomegaly, was likely a sign of ongoing fibrotic transformation of PV. Due to disease progression during ruxolitinib therapy, the medication could have been changed to fedratinib or, due to low platelet count, to pacritinib [32]. However, neither of these agents have been tested in this clinical setting. CMML-like progression of long-standing PV was excluded due to the fact that monocytosis was present from the very beginning. While the patient needed control over his Hct levels throughout the whole treatment period, the monocytosis was not progressive and cytopenias were not severe, therefore we did not address his condition from a CMML point of view. Treatment with azacitidine could have been an option for such an approach, however, ruxolitinib is also used in these instances with meaningful clinical activity [33]. In case of transformation to secondary myeloid leukaemia, a combination treatment including azacitidine and enasidenib could have been a reasonable option. This combination has been used in newly diagnosed and secondary leukaemia with *IDH2* mutation, achieving an overall response rate of 74% with favourable safety profile [34]. In our case, the MPN and LCH shared the same mutation in *IDH2*, both of which could be successfully targeted by enasidenib, as well as mutations in *ARAF*. Patients with histiocytic neoplasms and *ARAF* mutation are rarely reported and clinical experience with successful treatment is limited to cobimetinib which can achive up to 89% response rate in this patient population with durable responses [35, 36]. Vemurafenib could be a second-choice treatment option since it is effective in *ARAF* mutated histiocytic neoplasms in vitro [37]. If indicated and if the bone marrow reserve would have been adequate, the patient could have received standard chemotherapy with cladribine or cytarabine [38]. Lastly, bone marrow stem cell transplant (SCT) could potentially cure both, post-PV myelofibrosis and LCH. While SCT is standard of care for myelofibrosis, it is experimental in LCH. Eligible patients with LCH for this treatment have been considerably younger, children and young adults, and with less comorbidities, where they could still achieve long-term disease control [39].

In conclusion, we are presenting the first case of LCH that developed at the background of *ARAF/IDH2/ASXL1/JAK2* mutated PV and was undoubtedly clonally related to it, sharing the same mutations in *ARAF/IDH2* and *ASXL1*. We propose a model of branching evolution of PV and LCH from a clonal, *ARAF/IDH2/ASXL1* bearing precursor. Moreover, we discuss the ontogenesis and lineage plasticity, as well as provide a review of literature about clonally related LCH and underlying MPN. This confirmation, of a clonal relationship between two rare diseases and the description of their branching evolution, sheds a new light on the pathogenesis of secondary LCH and association between different cell types. Lastly, it could serve as the foundation of future research into the development of new therapeutic approaches.

## Data Availability

NA.
